# Management of neonatal sepsis at Muhimbili National Hospital in Dar es Salaam: diagnostic accuracy of C – reactive protein and newborn scale of sepsis and antimicrobial resistance pattern of etiological bacteria

**DOI:** 10.1186/s12887-014-0293-4

**Published:** 2014-12-05

**Authors:** Martha Franklin Mkony, Mucho Michael Mizinduko, Augustine Massawe, Mecky Matee

**Affiliations:** Department of Paediatrics and Child Health, Muhimbili National Hospital, Dar es Salaam, Tanzania; Epidemiology Fogarty Fellow, The Dartmouth-Boston University Fogarty AIDS International Training and Research Program, Boston University, Boston, MA USA; Department of Paediatrics and Child Health, School of Medicine, Muhimbili University of Health and Allied Sciences, Dar esSalaam, Tanzania; Department of Microbiology and Immunology, School of Medicine, Muhimbili University of Health and Allied Sciences, Dar esSalaam, Tanzania

**Keywords:** C – Reactive protein, Newborn scale of sepsis, Hematological markers, Neonatal sepsis

## Abstract

**Background:**

We determined the accuracy of Rubarth’s newborn scale of sepsis and C- reactive protein in diagnosing neonatal sepsis and assessed antimicrobial susceptibility pattern of etiological bacteria.

**Methods:**

This cross sectional study was conducted at Muhimbili National Hospital in Dar es Salaam, Tanzania between July 2012 and March 2013. Neonates suspected to have sepsis underwent physical examination using Rubarth’s newborn scale of sepsis (RNSOS). Blood was taken for culture and antimicrobial sensitivity testing, full blood picture and C – reactive protein (CRP) performed 12 hours apart. The efficacy of RNSOS and serial CRP was assessed by calculating sensitivity, specificity, negative and positive predictive values, receiver operating characteristics (ROC) analysis as well as likelihood ratios (LHR) with blood culture result used as a gold standard.

**Results:**

Out of 208 blood samples, 19.2% had a positive blood culture. Single CRP had sensitivity and specificity of 87.5% and 70.9% respectively, while RNSOS had sensitivity of 65% and specificity of 79.7%. Serial CRP had sensitivity of 69.0% and specificity of 92.9%. Combination of CRP and RNSOS increased sensitivity to 95.6% and specificity of 56.4%. Combination of two CRP and RNSOS decreased sensitivity to 89.1% but increased specificity to 74%. ROC for CRP was 0.86; and for RNSOS was 0.81.

For CRP the LHR for positive test was 3 while for negative test was 0.18, while for RNSOS the corresponding values were 3.24 and for negative test was 0.43.

Isolated bacteria were *Klebsiella spp* 14 (35%), *Escherichia coli* 12 (22.5%), *Coagulase negative staphlococci* 9 (30%), *Staphylococcus aureus *4 (10%), and *Pseudomonas spp* 1 (2.5%). The overall resistance to the WHO recommended first line antibiotics was 100%, 92% and 42% for cloxacillin, ampicillin and gentamicin, respectively. For the second line drugs resistance was 45%, 40%, and 7% for ceftriaxone, vancomycin and amikacin respectively.

**Conclusions:**

Single CRP in combination with RNSOS can be used for rapid identification of neonates with sepsis due to high sensitivity (95.6%) but cannot exclude those without sepsis due to low specificity (56.4%). Serial CRP done 12hrs apart can be used to exclude non-cases. This study demonstrated very high levels of resistance to the first-line antibiotics.

## Background

Adequate and timely diagnosis of neonatal sepsis remains an important challenge to the clinician especially in developing countries [1]. Blood culture, which is the gold standard for definitive diagnosis, takes at least 48 hours up to 6 days [2], by which time the infection may have progressed with consequences on the morbidity and mortality of the neonates [1,2].

Inflammatory markers such as procalcitonin, C – reactive proteins (CRP) and haematological indices have also been used in diagnosing neonatal sepsis [3-7].

The advantage of CRP includes its very low serum level in normal infants and rapid rise within 6 to 8 hours after the onset of sepsis [5,7-10]. Previous studies have shown that quantitative serial CRP levels 12 – 24 hours offer the most sensitive and reliable information [10-12]. And can therefore be used as an adjuvant tool to guide physicians [11,13,14].

Haematological scoring system (HSS) based on FBP, total leukocyte count, neutrophils and platelets have also been used to predict neonatal sepsis [3,7].

In resource limited settings, where blood culture is not routinely done, relatively inexpensive screening tools such as CRP and HSS can be utilized as a screening tools, potentially serving lives [6].

An additional challenge in the management of neonatal sepsis in most developing countries is a reliance on empirical use of antibiotics based on a recommended list of antibiotics, which are increasingly become ineffective owing to growing antimicrobial resistance [15-18].

In a bid to improve the management of neonatal sepsis at Muhimbili National Hospital, Dar es Salaam, we set to determine the efficacy of serial C – reactive protein taken 12 hours apart and newborn scale of sepsis as screening tools and antimicrobial susceptibility patterns of the etiological agents.

## Methods

### Study setting, design and participants

This was a prospective cross sectional study conducted at Muhimbili National Hospital (MNH) neonatal unit between July 2012 and March 2013. MNH is the National Referral Hospital and University Teaching Hospital with neonatal unit admitting an average of 20 neonates a day. A total of 208 neonates who met the WHO case definition for neonatal sepsis [19] were recruited consecutively. The sample size was determined using Epi info version 6.0 based on the prevalence of blood stream infection of 13.9% found by Bloomberg et al. [16] in the same hospital.

### Inclusion criteria

A neonate who met clinical criteria by WHO case definition for septicemia [1] was included. The clinical definition included any one of the following features

- History of difficulty feeding
- History of convulsions
- Movement only when stimulated
- Respiratory rate ≥60 breaths per minute
- Severe chest indrawing.
- Axillary temperature ≥37.5°C
- Axillary temperature ≤35.5°C
- Bulging anterior fontanelle,
- Signs of infection on the skin with pus spots and umbilicus pus spots

### Exclusion criteria

- Unwillingness of the parent or guardian to participate in the study
- Very sick children in decompensate state and requiring resuscitation
- Neonates with severe congenital malformation such as anencephaly

### Clinical assessment and laboratory investigations

#### Rubarth’s newborn scale of sepsis

This tool has two parts [19]. The first part includes physical examination of the patient has eight parameters with a total score of 35 points. The second part includes five laboratory parameters with a total score of 20 points. The total score from both parameters is 55. A neonate a total score of 10 or more, was considered to have sepsis.

#### Collection of blood samples

About 3.5 mls of venous blood was aseptically drawn from peripheral vein. Two mls were inoculated into Bacteralert paediatric blood culture bottle (BacT/Alert PF (Organon-Teknika Corp., Durham, N.C.). Another 1ml was used for measurement of CRP while 0.5 mls was used for full blood picture. About twelve hours later another 1 ml of blood was collected for a second CRP determination.

#### Full blood picture

For determination of full blood picture, blood samples were collected in vacutainers containing EDTA (Ethylene diamine tetra-acetic acid) and analysed by CELLDYNE 3700 (Abbott Laboratories. Abbott Park, Illinois, U.S.A.). Normal ranges were taken to be between 5000 and 30,000/ml for WBC, 1000 and 2000 for neutrophils, 150,000 and 450,000/ml for platelets. The extreme value on either side was suggestive of ongoing neonatal sepsis.

#### CRP determination

To determine CRP blood samples were centrifuged for separation of the serum within 60 minutes of blood collection and analysis was performed using COBRA 400/400 plus system (Roche Diagnostic limited, Switzerland). A value of more than 5 mg/l was considered to be associated with sepsis.

#### Blood culture

Blood culture bottles were incubated at 37°C temperature for 24 h after which aliquots were sub-cultured on solid agar plates; blood agar (Oxoid, UK) and MacConkey agar (Oxoid, UK) and chocolate agars (Oxoid, UK) for up 96 hours before being regarded as having no growth. Identification was based on microscopic characteristics, colonial characteristics, and Biochemical tests as described by Murray et al. [20], including VITEX (BioMerieux, France) and API 20E (BioMerieux, France). Gram negative organisms were identified by oxidase, Triple sugar Iron (TSI), sulphur indole and motility (SIM), urease, citrate test, VP and Methyl red test. Whereas Gram positive organisms were catalase reaction, coagulase test, DNase test and bile esculin test [20].

### Antimicrobial sensitivity testing

Antimicrobial susceptibility of isolates was determined using disk diffusion method according to Clinical Laboratory standard Institute [21]. Sensitivity testing was performed for antimicrobials which included ampicillin, cloxacillin and gentamicin which are used as first line antibiotics and ceftriaxone and vancomycin and amikacin which are used as second line drugs for treatment of neonatal sepsis at MNH. The concentration of the disks were as follows; ampicillin 10 μg, cloxacillin 5 μg gentamicin 10 μg, ceftriaxone 30 μg, amikacin 30 μg, vancomycin 30 μg. Results were recorded as resistant, intermediate and sensitive. During data analysis isolates showing intermediate resistance were categorized as being resistant.

### Statistical analysis

Statistical Package for Social Sciences (SPSS) version 17 was used for data entering, cleaning and analysis. Sensitivity, specificity, likelihood ratios of CRP and the Rubarth’s newborn scale were calculated using blood culture as Gold standard. Receiver operating characteristics (ROC curve) analysis was used to determine the cut off points for both Rubarth’s neonatal scale score and CRPs. The areas under the curves (AUC) were established and the difference between them was used to determine the better test. A p value of <0.05 was considered statistically significant.

### Ethical consideration

The ethics committee of the Muhimbili University of Health and Allied Sciences (MUHAS) approved the study. Informed written consents were obtained from parents/guardian prior to recruitment.

## Results

### Baseline characteristics

A total of 208 neonates were enrolled in this study, of whom 108 (51.9%) were male babies. Their median age was 5.6 days (1 – 28 days), and more than half (52.9%) were ≤4 days, and majority (81.7%) weighed ≥2.5 kg. Upon examination 67.3% of the participants had fever, 38.9% low muscle tone, and 79.8% were found to have fast breathing (Table 1).

**Table 1:** **Baseline demographic characteristics of the neonates enrolled in the study**

	**N = 208**	**Frequency (%)**
**Age group**		
0 – 3	109	52.9
4 – 28	99	47.6
**Sex**		
Male	108	51.9
Female	98	48.1
**Body weight**		
<1000	2	0.96
1 – 1.4	10	4.81
1.5 – 2.5	26	12.5
>2.5	170	81.7
**Clinical features**		
Fever	147	67.3
Low muscle tone	81	38.9
Fast breathing	166	79.8

### Isolated bacterial pathogens

**Figure 1:**
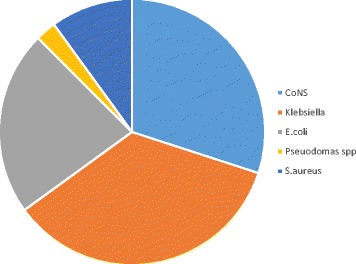
**Distribution of the isolated bacterial pathogens Figure** **2** **: Overall percentage resistance of isolated organisms to the recommended drugs.**

A positive blood culture was found in 40 (19.2%) of the 208 blood samples. The bacteria isolated included *Klebsiella spp *14 (35%), *E. coli* 12 (22.5%), *CoNS* 9 (30%), *S.aureas *4 (10%), and *Pseudomonas aeroginosa *1 (2.5%) (Figure 1).

### Antimicrobial sensitivity pattern of the isolated bacteria

**Figure 2:**
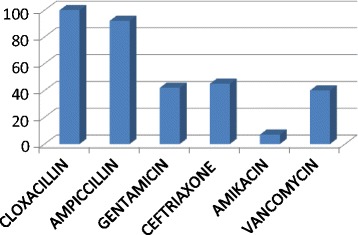
**Overall percentage resistance of isolated organisms to the recommended drugs.**

The overall resistance of isolated organisms to the recommended first line antibiotics for ampicillin was 92%, 100% to cloxacillin while gentamicin had moderate resistance of 42%. For the recommended second line antibiotics was 45% for ceftriaxone, 40% for vancomycin and 7% for amikacin (Figure 2).

### Rubarth’s neonatal scale of sepsis

RNSOS could identify 26 (65%) out of 40 neonates with positive blood cultures, while 79.8% of the 168 patients who had no growth on blood culture were correctly excluded by the test.

The likelihood ratio of the positive test was 3.24 and for negative test was 0.43 (Table 2).

**Table 2:** **Sensitivity, specificity, predictive values and likelihood ratios of CRP and RNSOS as a single test and when in combination**

	**Sensitivity (C.I)**	**Specificity (C.I)**	**PPV (C.I)**	**NPV (C.I)**	**LHR + ve test (C.I)**	**LHR - ve test (C.I)**	**P value**
**RNSOS**	65% (48 – 79.4)	79.7% (72.9 – 85.5)	43.3% (30.6 – 56.8)	90.5% (84.6 – 94.7)	3.24 (2.2 – 4.68)	0.45 (0.28 – 0.64)	<0.01
**CRP1**	87.5% (73.25 – 95.81)	70.8% (63.3 – 77.58)	41.7% (31 – 52.97)	95.9% (90.84 – 98.68)	3 (2.3 – 3.9)	0.18 (0.08 – 0.4)	<0.01
**CRP2**	78.9% (62.7 – 90.5)	75.8% (68 – 81.7)	42.3% (30.6 – 64.6)	93.9% (88.5 – 97.3)	3.2 (2.3 – 4.3)	0.27 (0.15 – 4.3)	<0.01
**CRP**_**1**_ **+ CRP**_**2**_	75.7% (58.8 – 88.2)	92.9% (87.9 – 96.2)	70% (53.4 – 83.4)	94.5 (89.9 – 97.4)	10.6 (4.1 – 17.1)	0.26 (0.10 – 0.42)	<0.01
**RNSOS + CRP**_**1**_	95.6% (90.0 – 97.5)	56.4% (38.7 – 70.7)	71% (51.9 – 85.7)	89.8 (84.4 – 93.8)	10.3 (2.7 –17.8)	0.47 (0.3 – 0.65)	0.01
**RNSOS + serial CRP**	47.5% (31.5 – 63.8)	96.4% (92.4 – 98.7)	76% (54.8 – 92.7)	88.5% (83 – 92.7)	13.3 (1.3 – 25.3)	0.54 (0.37 – 0.72)	0.03

C.I- confidence interval, CRP - C- reactive protein, RNSOS - Rubarth’s newborn scale of sepsis, PPV - Positive predictive value, NPV -negative predictive value, LHR - likelihood ratio.

### C reactive protein

#### First CRP

The first sample for CRP was positive in 35 (87.5%) out of 40 samples which had a positive blood culture and negative in 119 (70.8%) out of 168 samples with negative blood culture. The positive predictive value was 41.7% while negative predictive value was 95.9%. Likelihood ratio for positive test was 3 while for negative test was 0.18 (Table 2).

### Second CRP

Blood for the second sample for CRP (CRP_2_) was collected from all babies except for four who died before a sample was taken. CRP_2_ was positive in 30 (78.9%) out of 38 blood samples with a positive blood culture and was negative in 125 (75.3%) out of 166 blood samples with no bacterial growth. The positive predictive value of CRP_2_ was 42.3% while its negative predictive value was 93.9% (Table 2).

### Effect of combination of tests

Serial CRP had sensitivity of 69.0% and specificity of 92.9%. Combination of first CRP and RNOS had sensitivity of 95.6% while overall specificity decreased to 56.4%, while serial CRP and neonatal scale of sepsis had sensitivity of 89.1% and specificity was found to be 74.0% (Table 2).

### Receiver Operating Characteristics (ROC) analysis curve

**Figure 3:**
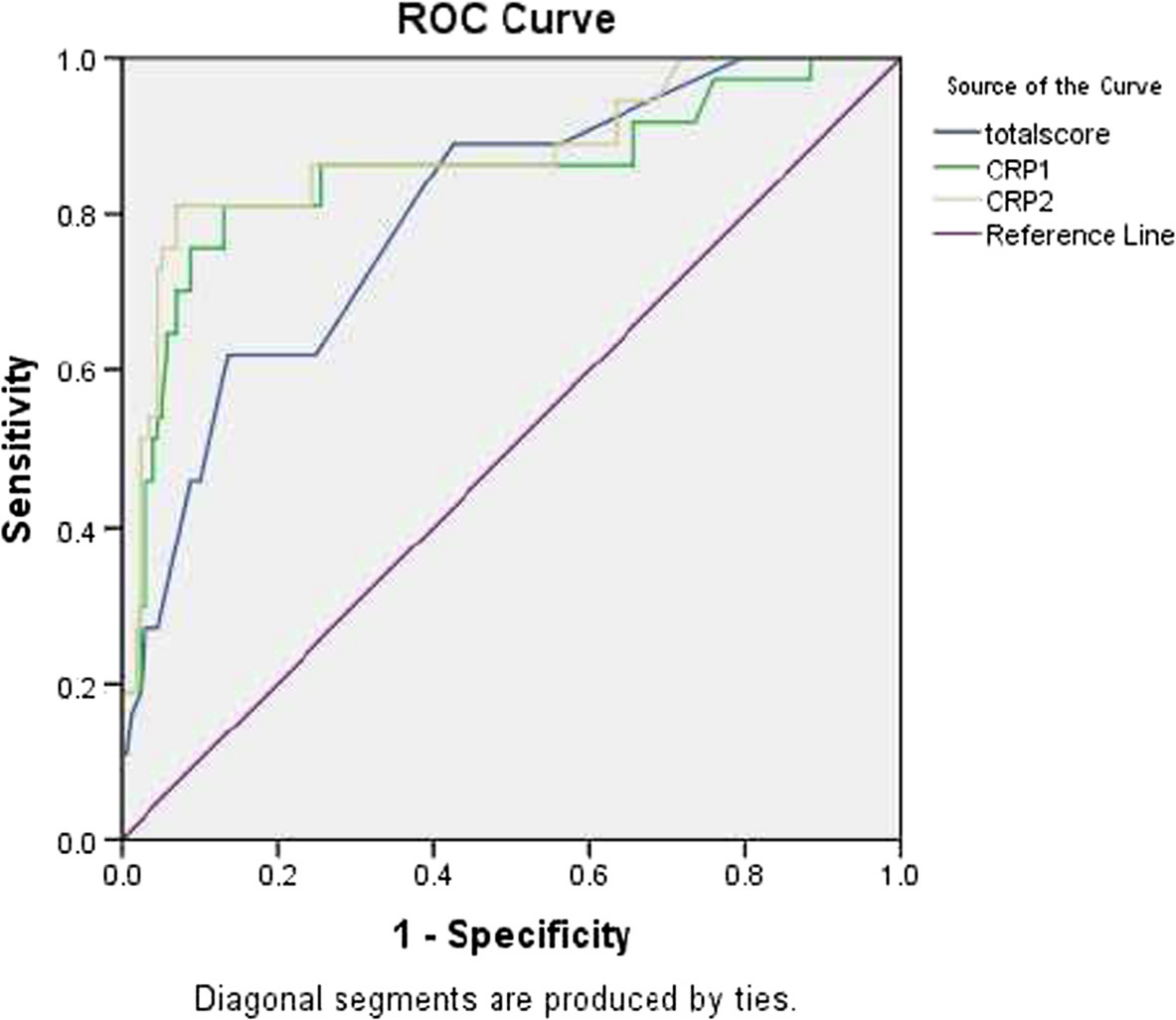
**ROC curve analysis for CRP**_**1**_ **, CRP**_**2**_ **and RNSOS.** ROC – receiver operating characteristic, CRP – C- reactive protein, RNSOS – Rubarth’s newborn scale of sepsis.

ROC analysis showed the following areas under the curve in determining septicaemia in neonates for CRP_1_ (AUC = 0.86; 95% CI: 0.78, 0.93) for CRP_2_ (AUC = 0.88; 95% CI: 0.80, 0.95) and for RNSOS vs. AUC = 0.81; 95% CI: 0.73, 0.88) (Figure 3). At optimal cut-off points for both CRP_1_ and CRP_2_ gave higher sensitivities than that of Rubarth’s scale (82.5% at the cut-off point 9 and 81.1% at the cut-off point 10 vs. 65% at cut-off point 10) (Figure 3).

## Discussion

We found a single CRP and RNSOS to have a very good sensitivity (96.0%) in identifying neonates with sepsis but had relatively low specificity (56.4%) in excluding non-cases. The positive and negative predictive values and likelihood ratio were also statistically significant showing the ability of the test to identity cases from suspected patients with neonatal sepsis.

Higher values are observed with quantitative analysis than qualitative CRP, and the range has been shown to be between 75 – 100% as demonstrated by studies done in Kenya by Kumar et al. [12], Ogunlesi et al. [22], west el al. [23] and Bomela et al. [13] in South Africa. Worth noting is the ROC analysis, the AUC for both test was very close to one encouragingly their use in resource poor settings where blood culture can not be done routinely. This finding has two implications; i) neonates with sepsis can quickly be identified and immediately started on treatment and thereby potentially reducing morbidity and mortality and ii) reduce unnecessary prescription of antibiotics and emergency of antibiotic resistance.

Based on our results we are suggesting a second CRP should be performed on neonates not picked by a combination of CRP and RNOS to exclude non-cases, and thus minimize the unnecessary antibiotics, and even those started treatment empirically can be stopped from antibiotic used [24,25].

We also found that the Rubarth’s score, which combines haematological parameters and physical parameters, had moderate sensitivity (79.7%) and specificity (65%) compared to when it was originally validated [21]. However when assessing the performance of individual parameters, leukopenia and thrombocytopenia were highly associated with sepsis thus indicating its usefulness in the diagnosis of sepsis.

The effect of combining CRP and RNSOS, had also a higher sensitivity than when RNSOS was used alone. Similar findings have been shown by Garland et al. [5] and Hengst et al. [4]. However Manucha et al. in India [6] concluded there was no advantage of combing hematological test with CRP in the management of neonatal sepsis. The controversy in findings can be due to the haematological score which was used. The added value demonstrated by our study can be because our scoring tool also assessed clinical presentation of the neonates.

From this study combination of the two tests have been proven to be effective and hence can be used for screening suspected cases of neonatal sepsis. Fortunately at MNH, FBP unlike blood culture, is done routinely and RNSOS which is inexpensive can be introduced. In a remote areas where some of the parameters of the RNSOS can not be done, using the ROC curve analysis has shown that CRP as either a single test or done serially has a better performance than RNSOS. The cost to process CRP sample at the time of study was an equivalent of approximately $10, compared to almost three times the cost to process blood culture excluding high level of expertise required for culture and sensitivity processing.

Proven neonatal sepsis by blood culture was found in only 19.2% of the neonates, which is in keeping with the findings of Bloomberg et al. in 2005 [16] and Mhada et al. in 2012 [15] at the same setting. The etiological agents that we found; *Klebsiella spp, E. coli, CoNS, S. aureus and Pseudomas spp* were similar to those reported by our colleagues [25-28], with minor variations.

The improper use of antibiotics maybe responsible for the very high levels of antibiotic resistance observed in this and previous studies conducted at this hospital [26,27]. Indeed, we found very high levels of resistance to the first line antibiotics, ampicillin and cloxaccilin in the range of 92 to 100%, with moderate resistance to gentamicin and ceftriaxone. The moderate resistance to ceftriaxone is due to its less frequent prescription since it is a second line drug and its relatively high cost compared to the first line antibiotics. Analysis of studies which were conducted between 1999 and 2012 at MNH and Bugando in Tanzania [15-18] has shown a gradual increase on the resistance not only with the recommended first line antibiotics but also to the alternative antibiotics vancomycin and amikacin. In this study however, we are seeing an alarming increase of resistance to amikacin when compared with the studies done by Bloomberg et al. [26] and in 2012 by Mhada et al. in the same setting [Table 3].

**Table 3:** **Trends of resistance to antibiotics used in management of neonatal sepsis**

**Drug**	**Blomberg et al. 2007 [** 26 ] (255*)	**Mhada et al. 2012 [** 15 **] (74)**	**Current study (40)**
Ampicillin	17%	88.2%	92%
Cloxacillin	12.5%	85.2%	100%
Gentamicin	37%	58.8%	42%
Ceftriaxone	Not done	16.2%	45%
Cefuroxime	17.6%	20.6%	Not done
Amikacin	Not done	1.5%	7%
Vancomycin	Not done	Not done	40%

*Included other paediatrics patients, out of which were 170 neonates.

The very high levels of resistance to the WHO recommended first line antibiotics have also been reported in other parts of the world. In Pakistan resistance levels as high as 100% to almost all the WHO recommended first line treatment, leading to a change of their treatment protocol [29].

## Conclusions

This study found that single CRP in combination with Rubarth’s neonatal scale of sepsis has a high sensitivity of 96.0% to screen for neonatal sepsis and serial CRP to be useful for excluding non-cases. We found out that only 19.2% of the neonates suspected of having sepsis actually required antibiotics*.* We speculate that excessive and unnecessary use of antibiotics has resulted in the very high levels of antibiotic resistance. We are advocating for the use of the inexpensive screening tools and for a change of the currently prescribed antibiotics
